# Frailty and Congestion in Patients with Heart Failure: Clinical Interaction and Prognostic Implications

**DOI:** 10.3390/jcm15124715

**Published:** 2026-06-17

**Authors:** Ángela Rodríguez-Eguren, Joan Llevadot-Sesmilo, José Jesús Broseta, Lydia Izquierdo, Eduard Solé-González, María Ángeles Castel, Juan José Rodriguez, Elena Cuadrado-Payán, Elena Sandoval, Marta Martínez-Chillarón, Diana Rodriguez-Espinosa, Aleix Cases, Francisco Maduell, Jose Maria Tolosana, Ana García-Álvarez, Marta Farrero, Pedro Caravaca-Pérez

**Affiliations:** 1 Cardiology Department, Institut Clínic Cardiovascular (ICCV), Hospital Clinic de Barcelona, Universitat de Barcelona, C/Villarroel 170, 08036 Barcelona, Spain; arodrigueze@clinic.cat (Á.R.-E.);; 2 Institut d’Investigacions Biomèdiques August Pi i Sunyer (IDIBAPS), 08036 Barcelona, Spaindmrodriguez@clinic.cat (D.R.-E.); fmaduell@clinic.cat (F.M.); 3 Nephrology and Renal Transplantation, Hospital Clinic de Barcelona, Universitat de Barcelona, C/Villarroel 170, 08036 Barcelona, Spain; 4 Laboratori Experimental de Nefrologia i Trasplantament (LENIT), 08036 Barcelona, Spain; 5 Department of Medicine, Universitat de Barcelona, 08007 Barcelona, Spain; 6 Heart Failure and Heart Transplant Unit, Institut Clínic Cardiovascular (ICCV), Hospital Clinic de Barcelona, Universitat de Barcelona, C/Villarroel 170, 08036 Barcelona, Spain; 7 Centro de Investigación Biomédica en Red, Enfermedades Cardiovasculares (CIBERCV), 28029 Madrid, Spain; 8 Cardiovascular Surgery Department, Hospital Clinic de Barcelona, Universitat de Barcelona, C/Villarroel 170, 08036 Barcelona, Spain; 9 Nephrology, Hospital Moisès Broggi, 08970 Sant Joan Despí, Spain; 10 Centro Nacional de Investigaciones Cardiovasculares (CNIC), 28029 Madrid, Spain

**Keywords:** heart failure, congestion, frailty

## Abstract

**Background/Objectives:** Frailty and congestion are highly prevalent in patients with heart failure (HF) and are independently associated with adverse outcomes. However, their interrelationship and combined prognostic significance remain incompletely understood. We aimed to investigate the association between frailty and congestion phenotypes in older patients with HF and to evaluate their combined impact on clinical outcomes. **Methods:** We prospectively included 308 ambulatory patients aged ≥65 years with chronic HF from a specialized HF clinic between 2022 and 2024. Frailty was assessed using the Fried phenotype. Congestion was defined by clinical signs and/or elevated NT-proBNP (≥1000 pg/mL) and CA125 (≥35 U/mL). Associations between congestion and frailty were evaluated using logistic regression and restricted cubic spline analyses. The primary endpoint was a composite of all-cause mortality or HF hospitalization at 1 year. **Results:** Frailty was present in 49.7% of patients and congestion in 79.9%. Frailty prevalence increased progressively across congestion phenotypes, from 21.0% in patients without congestion to 70.8% in those with combined clinical and biochemical congestion (*p* < 0.001). Congestion was also associated with worse nutritional status and reduced muscle strength. In multivariable analyses, frailty and congestion remained independently associated with adverse outcomes. Their coexistence identified the subgroup with the highest risk of death or HF hospitalization (HR 25.54, 95% CI 3.46–188.26; *p* = 0.001). **Conclusions:** In older ambulatory patients with HF, frailty and congestion frequently coexisted and identified patients at increased risk of adverse outcomes, particularly when clinical and biochemical congestion were present. These findings support combining frailty evaluation with congestion assessment for prognostic stratification.

## 1. Introduction

Heart failure (HF) is a complex clinical syndrome associated with substantial morbidity and mortality [1]. Beyond cardiac dysfunction, HF is increasingly recognized as a systemic condition characterized by reduced physiological reserve and vulnerability to stressors, a state commonly reflected by frailty [2]. Frailty affects approximately half of patients with HF and is particularly prevalent in specific phenotypes, such as HF with preserved ejection fraction [2]. The condition arises from mechanisms including inflammation, sarcopenia, and physical inactivity, and is consistently associated with worse outcomes, conferring a 1.5–2-fold higher risk of mortality and hospitalization [3].

Congestion, another hallmark of HF, is a major driver of clinical decompensation and hospital admission [4,5]. Congestion may manifest as elevated cardiac filling pressures (intravascular congestion) or interstitial fluid accumulation (tissue congestion), two often overlapping entities [6,7]. Its identification remains challenging, as clinical assessment alone may underestimate its true burden, supporting the use of multimodal approaches incorporating biomarkers such as NT-proBNP and CA125. Importantly, both residual congestion at discharge and greater severity at admission are associated with an increased risk of rehospitalization and mortality [5,6].

Although frailty and congestion are well-established predictors of adverse outcomes in HF, their interrelationship remains poorly defined. Most studies have evaluated these conditions separately, despite their potential biological overlap and frequent coexistence in clinical practice. However, it remains unclear to what extent congestion burden is associated with the presence and severity of frailty, whether specific congestion phenotypes are more strongly linked to frailty, and whether their coexistence identifies patients at particularly high risk.

Accordingly, the present study sought to characterize the relationship between congestion and frailty in patients with HF and to evaluate their combined prognostic impact, with the goal of improving risk stratification and informing the management of this vulnerable population.

## 2. Materials and Methods

### 2.1. Study Design and Population

Consecutive patients were prospectively recruited from a specialized HF clinic at Hospital Clínic of Barcelona between December 2022 and December 2024. Eligible patients had a confirmed diagnosis of chronic HF according to European Society of Cardiology criteria [8].

All patients were managed according to guideline-directed medical therapy when appropriate and were followed in a dedicated HF clinic. The study protocol was approved by the institutional ethics committee, and all participants provided written informed consent in accordance with the Declaration of Helsinki.

### 2.2. Data Collection

Baseline demographic, clinical, and laboratory data were prospectively collected from electronic medical records. Laboratory parameters included complete blood count, renal function, electrolytes, NT-proBNP, CA125, and spot urinary indices, including albumin-to-creatinine ratio and urinary sodium. Echocardiographic data, including left ventricular ejection fraction (LVEF), were obtained from the most recent echocardiographic assessment within the preceding 12 months, reflecting routine clinical practice.

### 2.3. Definitions and Baseline Variables

Frailty was defined according to the Fried physical phenotype [9], which includes five components: unintentional weight loss, weakness, exhaustion, slow gait speed, and low physical activity. Each component was assessed dichotomously (present/absent), generating a score from 0 to 5. Weakness was evaluated by handgrip strength using sex- and body mass index-adjusted cut-offs, while slowness was determined through a standardized 4-m gait speed test with sex- and height-specific thresholds. Unintentional weight loss was defined as a self-reported loss of ≥4.5 kg or ≥5% of body weight during the previous year. Exhaustion was identified from patient-reported fatigue and reduced energy during daily activities using standardized questions. Low physical activity was assessed according to a reduction in habitual physical activity levels based on structured self-report assessment. Patients fulfilling ≥3 criteria were classified as frail, whereas those with 0–2 criteria were categorized as non-frail/prefrail and analyzed as a single reference group to improve statistical power and model stability. Although prefrailty may represent a clinically distinct phenotype, both groups were combined to preserve statistical power and model stability given the sample size.

Nutritional status was assessed using the Mini Nutritional Assessment–Short Form (MNA-SF), a validated screening tool specifically designed for older adults [10]. The instrument comprises six domains evaluating reduced food intake, recent weight loss, mobility, psychological stress or acute illness, neuropsychological disorders, and body mass index (or calf circumference when BMI was unavailable). Total scores range from 0 to 14, with established cut-offs indicating normal nutritional status (12–14 points), risk of malnutrition (8–11 points), and malnutrition (0–7 points).

Muscle strength was measured by handgrip dynamometry using sex- and body mass index–specific thresholds based on the original Fried criteria [9]. Additional geriatric domains, including functional status and comorbidity burden, were evaluated using validated instruments [11,12,13].

Congestion was defined as clinical and/or biochemical evidence of fluid overload [4]. Clinical congestion was defined by the presence of at least one of the following: peripheral edema, elevated jugular venous pressure, or ascites. Biochemical congestion was defined by elevated circulating biomarkers, including NT-proBNP and/or CA125, using thresholds of ≥1000 pg/mL [14] and ≥35 U/mL [15], respectively. In addition, a pathophysiological classification based on biomarker profiles was applied, distinguishing intravascular congestion (NT-proBNP ≥ 1000 pg/mL only), extravascular congestion (CA125 ≥ 35 U/mL only), combined congestion (both biomarkers elevated), and no congestion when neither criterion was met.

As an exploratory sensitivity analysis, a lower NT-proBNP threshold (≥500 pg/mL) was applied in patients with advanced CKD (eGFR 15–45 mL/min/1.73 m^2^), consistent with recent evidence in HF and CKD populations [16].

LVEF was measured using the biplane Simpson method and classified as preserved (≥50%), mildly reduced (40–49%), or reduced (≤40%) [8].

Renal function was estimated using the Chronic Kidney Disease Epidemiology Collaboration (CKD-EPI) creatinine and cystatin C equations [17].

Missing data were handled using a complete-case approach without imputation.

### 2.4. Outcomes and Follow-Up

Patients were followed for 365 days. The primary endpoint was a composite of all-cause mortality and hospitalization for HF. For time-to-event analyses, the event date was defined as the date of the first occurring event, either death or HF hospitalization. Events were identified from clinical records and adjudicated by independent investigators blinded to frailty and congestion status.

### 2.5. Statistical Analysis

Normality was assessed using the Shapiro–Wilk test. As most variables were non-normally distributed, continuous data are presented as median (interquartile range [IQR]) and compared using the Mann–Whitney U test. Categorical variables are reported as n (%) and compared using the χ^2^ test or Fisher’s exact test, as appropriate.

Associations between congestion and frailty were evaluated using logistic regression. The relationship between congestion biomarkers (NT-proBNP and CA125) and frailty was further examined using restricted cubic spline models. Biomarkers were natural log-transformed to account for skewed distributions. Predicted probabilities were plotted across the biomarker range with 95% confidence intervals. Odds ratios were estimated using median values as the reference (NT-proBNP: 2420 pg/mL; CA125: 15 U/mL).

Time-to-event analyses were performed using Kaplan–Meier estimates and compared using the log-rank test. Cox proportional hazards models were used to assess the association between frailty, congestion, and outcomes. Multivariable models were constructed based on clinical relevance and prior evidence, with additional inclusion of variables showing a *p*-value < 0.10 in univariable analysis. Final models were derived using backward stepwise selection and included age, sex, frailty, and congestion. Model discrimination was assessed using Harrell’s C-statistic, and the proportional hazards assumption was tested using Schoenfeld residuals. Due to the small sample size and the resulting risk of unstable estimates, non-frail patients with isolated biochemical congestion were excluded from the forest plot representation but were retained in all regression analyses.

A two-sided *p*-value < 0.05 was considered statistically significant. All analyses were performed using Stata 19 (StataCorp, College Station, TX, USA).

Generative artificial intelligence (GenAI) tools (ChatGPT-5.5) were used during manuscript preparation to improve language and readability. The authors reviewed and edited the generated output and were responsible for all scientific content, data analyses, interpretation of results, and conclusions.

## 3. Results

### 3.1. Baseline Characteristics

**Figure 1:**
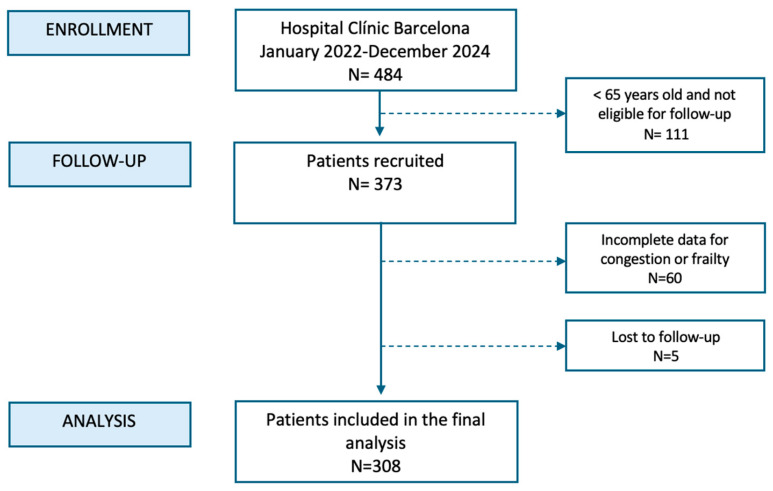
Flow diagram of the study population. A total of 484 consecutive patients from the outpatient heart failure clinic at Hospital Clínic Barcelona between January 2022 and December 2024 were initially screened. Of these, 111 were excluded due to age < 65 years or ineligibility for follow-up, mainly because of dementia or an estimated life expectancy <1 year. A total of 373 patients were recruited. During follow-up, 60 patients were excluded due to incomplete data on congestion or frailty assessment. Congestion was defined using clinical variables in combination with NT-proBNP and CA125 levels, whereas frailty was assessed using the Fried phenotype. Additionally, 5 patients were lost to follow-up. The final study population comprised 308 patients enrolled in the analysis.

A total of 484 patients were screened, of whom 308 met the inclusion criteria after excluding those with incomplete frailty or congestion data (Figure 1).

Baseline characteristics of the overall study population are shown in Table 1. The cohort consisted of older patients with a high burden of comorbidity, advanced cardiorenal disease, and a high prevalence of both frailty and congestion.

#### 3.1.1. Frailty

Frailty was present in 49.7% of the cohort, while 35.4% were prefrail and 14.9% non-frail, yielding a combined prevalence of 50.3% for prefrail/non-frail status. The cohort exhibited a high comorbidity burden, with all patients having a Charlson Comorbidity Index ≥ 3. Severe disability according to the Barthel Index was present in 24 patients (7.8%), while cognitive impairment based on the Pfeiffer scale was observed in 19 patients (6.3%). Baseline characteristics according to frailty status are shown in Table 1.

#### 3.1.2. Congestion

Congestion was highly prevalent in the cohort (79.9%) and was associated with a more adverse clinical profile, including older age, worse renal function, and markers of greater cardiorenal impairment (Table 2).

When frailty and congestion were considered jointly, the coexistence of both conditions identified a subgroup of patients with the most adverse clinical profile, as shown in Supplementary Table S1.

### 3.2. Relationship Between Congestion Phenotypes and Frailty

**Figure 2:**
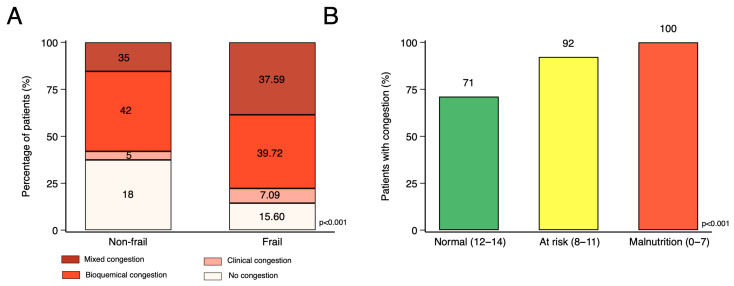
Congestion profiles and their relationship with frailty and nutritional status. (**A**) Congestion profiles according to frailty status. Stacked bars represent the distribution of congestion phenotypes stratified by frailty. Light gray indicates no congestion, light red clinical congestion, red biochemical congestion, and dark red both clinical and biochemical congestion. Clinical congestion was defined as the presence of jugular venous distension, peripheral edema, or ascites, while biochemical congestion was defined as NT-proBNP > 1000 pg/mL or CA125 > 35 U/mL. (**B**) Prevalence of congestion according to nutritional status as defined by the Mini Nutritional Assessment Short Form (MNA-SF) classification. Nutritional categories are color-coded as follows: normal (12–14; green), at risk of malnutrition (8–11; yellow), and malnutrition (0–7; orange/red). The proportion of patients with congestion increased progressively across worsening nutritional status (*p* < 0.001).

Congestion correlated with frailty, with a higher prevalence among patients with congestion than in those without (91.5% vs. 68.4%; *p* < 0.001). As shown in Figure 2A, the prevalence of frailty showed a stepwise increase across congestion phenotypes, from 21.0% in patients without congestion to 70.8% in those with both clinical and biochemical congestion (*p* < 0.001). Non-frail patients more frequently presented without congestion (31.6% vs. 8.5%). In a sensitivity analysis preserving the original Fried frailty categories, the prevalence of congestion increased progressively from robust to prefrail and frail patients (43.5%, 78.9%, and 91.5%, respectively; *p* < 0.001), supporting the existence of a graded relationship between congestion burden and frailty severity (Supplementary Table S3).

**Figure 3:**
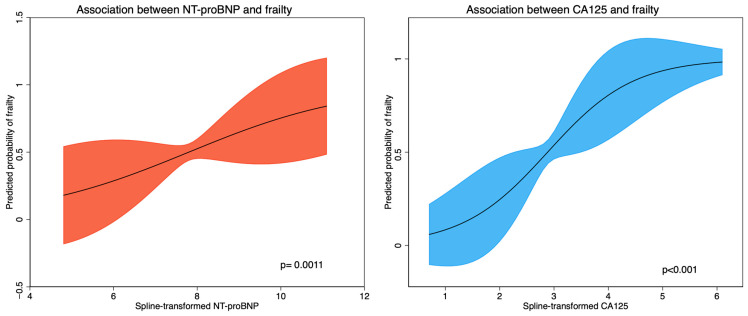
Association between NT-proBNP and CA125 and frailty using restricted cubic spline models. Curves represent the predicted probability of frailty across the range of each biomarker, with shaded areas indicating 95% confidence intervals. Models were adjusted for age, sex, and eGFR.

At the biomarker level, both NT-proBNP and CA125 were associated with frailty. Restricted cubic spline analyses demonstrated a linear relationship between these biomarkers and the probability of frailty (*p* for overall association = 0.001 and *p* < 0.001, respectively), with no evidence of non-linearity (*p* = 0.35 and *p* = 0.63, respectively) (Figure 3).

In univariable logistic regression analysis, all congestion profiles were associated with higher odds of frailty compared with no congestion. The strongest association was observed when both clinical and biochemical congestion were present (OR 9.13, 95% CI 4.26–19.59; *p* < 0.001), followed by clinical congestion (OR 6.03, 95% CI 1.69–21.55; *p* = 0.006) and biochemical congestion (OR 3.47, 95% CI 1.73–6.94; *p* < 0.001). Results of the univariable and multivariable logistic regression analyses for determinants of frailty and congestion are shown in Supplementary Table S2.

Congestion was also linked to markers of systemic vulnerability. As illustrated in Figure 2, this relationship extended beyond frailty to nutritional status. Patients with congestion exhibited worse nutritional status (*p* < 0.001), with a stepwise gradient across MNA-SF categories: 64.2% in patients with normal nutritional status, 87.4% in those at risk, and 100% in malnourished patients (*p* < 0.001) (Figure 2B). In multinomial analysis, congestion was associated with an increased risk of being in the “at risk of malnutrition” category compared with normal nutritional status (RRR 3.86, 95% CI 2.01–7.43; *p* < 0.001).

Similarly, congestion was associated with markers of sarcopenia. The prevalence of reduced muscle strength was higher among patients with congestion (82.8% vs. 55.6%; *p* < 0.001), and this association remained significant in multivariable analysis (OR 3.13, 95% CI 1.70–5.76; *p* < 0.001).

In the sensitivity analysis using an alternative NT-proBNP threshold in patients with advanced chronic kidney disease, 14 patients (4.5% of the cohort) were reclassified, resulting in an increase in congestion prevalence from 79.9% to 84.4%. Despite this reclassification, the associations of congestion with frailty and clinical outcomes remained materially unchanged (Supplementary Table S4).

### 3.3. Prognostic Impact of Frailty and Congestion

**Figure 4:**
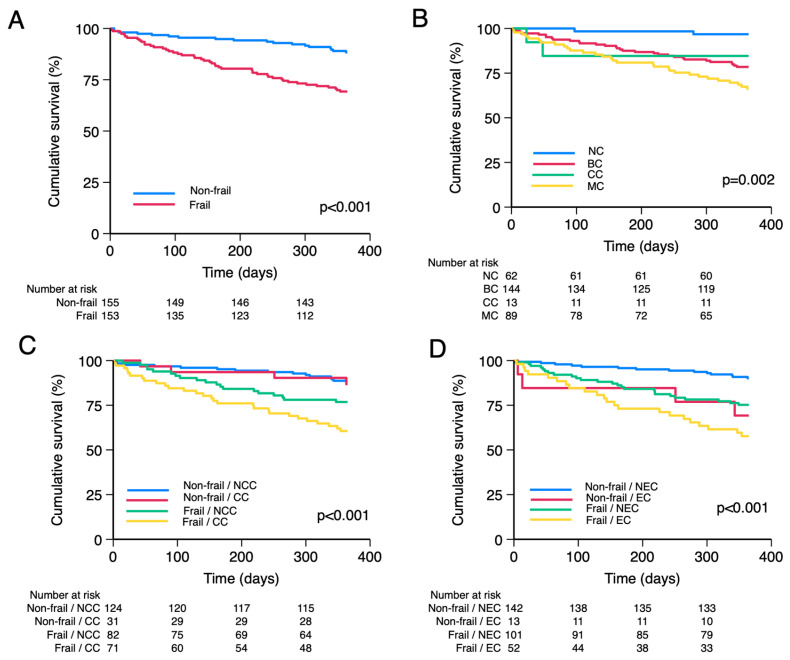
Kaplan–Meier survival curves according to frailty and different congestion phenotypes. (**A**) Survival according to frailty status (frail vs. non-frail). (**B**) Survival according to congestion type: no congestion (NC), biochemical congestion (BC), clinical congestion (CC), and mixed biochemical and clinical congestion (MC). (**C**) Survival according to the combination of frailty and clinical congestion: non-frail/no clinical congestion (NCC), non-frail/clinical congestion (CC), frail/no clinical congestion (NCC), and frail/clinical congestion (CC). (**D**) Survival according to frailty and extracellular congestion assessed by CA125: non-frail/no extracellular congestion (NEC), non-frail/extracellular congestion (EC), frail/no extracellular congestion (NEC), and frail/extracellular congestion (EC). *p* values were calculated using the log-rank test.

During 365 days of follow-up, 65 events were recorded, including 42 hospitalizations for HF and 37 deaths. Kaplan–Meier curves demonstrated progressively worse event-free survival according to both frailty status and congestion phenotype, with the poorest prognosis observed in patients with both conditions (Figure 4).

In univariable analysis, frailty was associated with worse outcomes (HR 3.00, 95% CI 1.74–5.17; *p* < 0.001), as was congestion (HR 8.98, 95% CI 2.20–36.70; *p* = 0.002) (Table 3). When stratified by congestion phenotype, a graded increase in risk was identified, with the highest risk in patients with coexisting clinical and biochemical congestion (HR 12.38, 95% CI 2.96–51.80; *p* = 0.001), followed by isolated biochemical congestion (HR 7.35, 95% CI 1.76–30.72; *p* = 0.006), whereas isolated clinical congestion was not significantly associated with outcomes (HR 5.38, 95% CI 0.76–38.21; *p* = 0.092).

**Figure 5:**
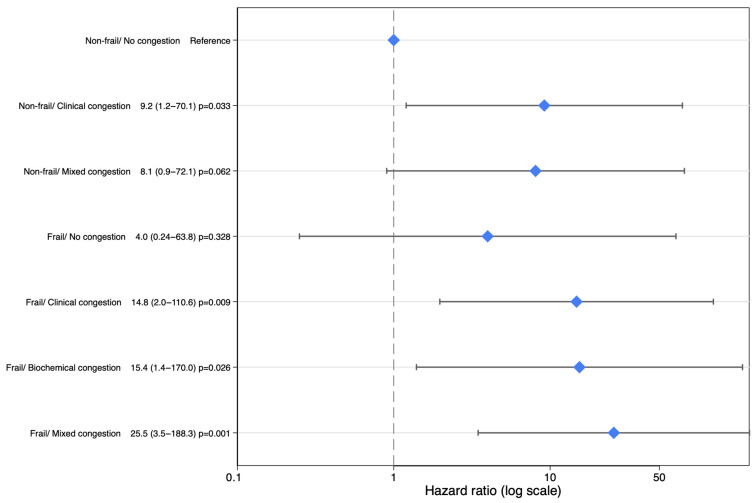
Prognostic impact of combined frailty and congestion phenotypes on 1-year outcomes. Forest plot showing hazard ratios (HR) and 95% confidence intervals (CI) for the association between combined frailty and congestion phenotypes and the risk of the primary outcome at 1 year. The reference group was patients without frailty and without congestion. Congestion phenotypes were classified as clinical or biochemical. Frailty was assessed as a binary variable (frail vs. non-frail), and combined with congestion status to generate six distinct groups. All estimates were derived from Cox proportional hazards regression models. The dashed vertical line indicates the reference value (HR = 1). Results are presented on a logarithmic scale. Due to the very low sample size and unstable estimates, the subgroup ‘non-frail with isolated biochemical congestion’ is not displayed in the figure, although it was included in the regression model.

A similar pattern emerged using the pathophysiological classification. Concomitant intravascular and extravascular congestion remained the strongest predictor (HR 9.37, 95% CI 3.27–26.87; *p* < 0.001), followed by isolated intravascular congestion (HR 4.18, 95% CI 1.47–11.77; *p* = 0.007), whereas isolated extravascular congestion was not associated with outcomes. When frailty and congestion phenotypes were analyzed jointly, a clear risk gradient was observed, with the highest event rates among frail patients with biomarker-defined combined congestion (HR 25.54, 95% CI 3.46–188.26; *p* = 0.001), a finding confirmed by Cox regression analyses (Figure 5).

In multivariable analysis adjusted for age and sex, both frailty and congestion remained independently associated with outcomes (Table 3). Inclusion of congestion improved model discrimination, increasing Harrell’s C-statistic from 0.62 to 0.67. The proportional hazards assumption was satisfied for all variables (global test *p* = 0.56).

Results were consistent in a competing-risk sensitivity analysis evaluating HF hospitalization with all-cause mortality as a competing event. Congestion remained independently associated with a higher risk of HF hospitalization (subdistribution HR 9.55, 95% CI 1.30–70.00; *p* = 0.026) (Supplementary Table S5).

## 4. Discussion

In this study of patients with HF, we sought to characterize the relationship between frailty and congestion and to evaluate their combined prognostic impact. Four principal findings emerge. First, both frailty and congestion were highly prevalent and identified a subgroup of patients with a more advanced and complex clinical profile. Second, a clear and graded association was observed between the two conditions: congestion was markedly more frequent among frail individuals, and the prevalence of frailty increased progressively across more severe congestion phenotypes, particularly in the presence of coexisting clinical and biochemical congestion. Third, congestion was associated not only with frailty itself but also with key markers of physical vulnerability, including impaired nutritional status and reduced muscle strength. Fourth, both frailty and congestion were independently associated with adverse outcomes at 1 year, with the highest risk observed when both conditions coexisted. Collectively, these findings indicate that frailty and congestion represent complementary yet closely related dimensions of vulnerability in HF and support the hypothesis that the systemic effects of congestion may contribute to the development of frailty.

### 4.1. Frailty

Frailty is an established marker of vulnerability in patients with HF and is associated with increased morbidity and mortality compared with non-frail individuals. [3] In our cohort, frailty was present in approximately half of the patients, consistent with previous studies using the Fried frailty phenotype, which have reported prevalence rates ranging from 19% to 52% in patients with HF. [3] Differences in HF therapy according to frailty status were also observed; however, these findings should be interpreted in the context of the differing LVEF distribution between groups, particularly the higher prevalence of preserved LVEF among frail patients, which may have influenced the prescription of guideline-directed medical therapies.

These findings reinforce the concept that frailty in HF reflects the cumulative burden of multisystem impairment rather than chronological aging alone.

### 4.2. Congestion

Congestion affected nearly 80% of the cohort, a figure consistent with previous studies in ambulatory HF populations reporting rates of 58–71% when assessed using a multimodal approach, including imaging techniques [7]. In contrast, only 41% of patients in those series exhibited overt clinical signs of congestion, a proportion comparable to the prevalence of clinical congestion observed in our cohort (33.1%). These findings support the concept that reliance on physical examination alone may fail to identify a substantial proportion of patients with congestion, underscoring its limited sensitivity. In this context, the use of integrated diagnostic strategies, including imaging techniques or circulating biomarkers such as natriuretic peptides and CA125, may enable more accurate identification of congestion.

Within this framework, biomarker-based assessment requires careful interpretation. Natriuretic peptide levels may be influenced by factors such as obesity, atrial fibrillation, and renal dysfunction [18], whereas CA125 may be less reliable in certain subgroups, including patients with preserved ejection fraction, and may be affected by confounding conditions such as malignancy [19,20]. Nonetheless, their accessibility and ability to detect subclinical congestion support their use as complementary tools in clinical practice.

Importantly, congestion was associated with a pattern of multisystem impairment involving renal dysfunction and adverse nutritional markers. Although several mechanisms potentially linking congestion to organ dysfunction have been proposed—including impaired organ perfusion, venous congestion, and inflammatory activation related to splanchnic congestion [3]—the cross-sectional design precludes establishing the directionality or causal contribution of these associations. Nevertheless, these findings suggest that congestion frequently coexists with a more clinically complex phenotype.

The higher prevalence of hyponatraemia observed among congested patients is also consistent with greater neurohormonal activation and cardiorenal dysfunction. In this regard, recent evidence suggests that electrolyte-derived indices, such as the sodium-to-chloride ratio, may provide additional prognostic information in patients with HF, although their role in chronic ambulatory HF populations remains to be fully established [21].

### 4.3. Interplay Between Congestion and Frailty

A central observation of the present study is the graded relationship between congestion and frailty. Frail patients were progressively overrepresented across increasing levels of congestion, particularly among those with coexisting clinical and biochemical congestion, suggesting a continuum in which greater congestion burden parallels greater biological vulnerability. Congestion was also associated with a higher risk of malnutrition and reduced muscle strength, consistent with a relationship that extends beyond symptom burden alone and involves key determinants of physical function.

These findings raise the possibility that congestion may contribute to the development or progression of frailty in HF. Such an association is biologically plausible, as the systemic consequences of congestion—including neurohormonal activation, inflammation, and end-organ dysfunction—may adversely affect nutritional status, skeletal muscle function, and physical performance. Previous studies support this conceptual framework. Biomarkers such as growth differentiation factor-15 (GDF-15) have been associated with both greater congestion and the presence of anorexia/cachexia [22], suggesting a common underlying biological axis. Similarly, intestinal congestion has been associated with gastrointestinal symptoms, appetite loss, and cachexia, while bowel wall thickness and right atrial pressure have remained independently associated with cachexia after adjustment [23]. Regarding inflammation, despite growing evidence supporting a role for immune activation in both HF progression and frailty development, its precise contribution remains incompletely understood [21]. Since inflammatory biomarkers were not systematically assessed in the present study, the contribution of inflammation to the observed association between congestion and frailty cannot be directly evaluated and remains speculative.

Taken together, these observations suggest that frailty may represent a clinically measurable manifestation of the systemic consequences of congestion, providing a potential link between underlying pathophysiological derangements and adverse clinical outcomes.

### 4.4. Prognostic Implications of Frailty and Congestion

Both frailty and congestion were associated with worse 1-year outcomes and remained independently associated with events after adjustment for age and sex. Furthermore, the inclusion of congestion improved model discrimination, supporting its incremental prognostic value beyond traditional clinical variables.

Patients with both clinical and biochemical congestion showed the strongest association with outcomes, whereas isolated clinical congestion was not significantly associated with prognosis. This finding should be interpreted with caution, however, as the small number of patients with isolated clinical congestion may have limited statistical power. Nevertheless, these results may indicate that reliance on clinical signs alone could underestimate congestion burden and fail to identify patients at increased risk.

Given the independent associations of both congestion and frailty with adverse outcomes, together with the relationship between congestion and markers of physical decline, it is plausible that effective decongestive strategies may have clinically relevant implications, particularly in frail patients. This hypothesis warrants prospective evaluation. In this context, analyses from the FINEARTS-HF trial suggest a greater absolute benefit of finerenone among patients with a higher frailty burden [24], although this observation should be interpreted with caution.

From a practical perspective, these findings underscore the importance of systematic congestion assessment in older patients with HF and support a more integrated cardiorenal and geriatric approach to follow-up, in which congestion assessment and frailty screening are viewed as complementary components of patient evaluation.

Several limitations merit consideration. First, this was an observational study, and causal inferences cannot be established. Although the data support a robust association between congestion and frailty, the directionality of this relationship remains uncertain, as frailty itself may contribute to congestion through reduced mobility, impaired self-care, or delayed recognition of decompensation. Second, congestion was defined using clinical signs and circulating biomarkers without direct hemodynamic measurements or systematic imaging assessment, which may limit comparability with other studies and may not fully capture the complexity of congestion. In addition, the high prevalence of renal dysfunction in the cohort may have influenced biomarker levels, particularly NT-proBNP, potentially leading to overestimation of biochemical congestion. Third, the study population consisted of older patients recruited from a tertiary heart failure and cardiorenal referral clinic, with a high burden of comorbidity and a high prevalence of advanced renal dysfunction. Consequently, the study cohort represents a selected population with greater clinical complexity than that typically encountered in community-based heart failure settings. Therefore, the prevalence of frailty, congestion burden, and their association with outcomes may differ in younger patients or in less complex heart failure populations, limiting the external generalizability of our findings. Moreover, substantial clinical overlap likely exists among frailty, malnutrition, sarcopenia, and congestion in older patients with HF, making it difficult to fully disentangle the independent contribution of each condition. Consequently, part of the observed prognostic effect may reflect shared underlying pathophysiological mechanisms rather than distinct clinical entities. Inflammatory biomarkers were not systematically measured; therefore, the potential contribution of inflammation to the observed association between congestion and frailty could not be directly evaluated. Fourth, some subgroup analyses included relatively small numbers of patients, resulting in wide confidence intervals and potentially unstable estimates, particularly in less frequent congestion phenotypes and joint frailty–congestion categories. Finally, frailty was assessed only at baseline, precluding evaluation of its temporal evolution, potential reversibility, or the impact of decongestive therapies on frailty trajectories over time.

In older patients with HF, frailty and congestion appear to represent closely interconnected manifestations of disease complexity rather than isolated clinical entities. The progressive increase in frailty across congestion phenotypes, together with the associations of congestion with impaired nutritional status, reduced muscle strength, and adverse outcomes, suggests a close interplay between these conditions. Considered together, they may provide a more comprehensive assessment of patient status than either condition alone.

## 5. Conclusions

In this cohort of older ambulatory patients with HF, frailty and congestion were highly prevalent and frequently occurred together, identifying a subgroup at increased risk of adverse outcomes, particularly in the presence of simultaneous clinical and biochemical congestion. These findings highlight the close relationship between frailty and congestion and suggest that combining routine frailty assessment with multimodal congestion evaluation may provide a simple and clinically feasible approach to risk stratification in this population. Future studies are needed to better elucidate the mechanisms underlying this interplay and evaluate whether interventions targeting congestion can modify frailty trajectories and ultimately improve clinical outcomes.

## Figures and Tables

**Table 1:** Baseline Characteristics of the Study Population according to frailty status. The first column shows the overall study population, and subsequent columns present data according to the presence or absence of frailty. Continuous variables are presented as median (interquartile range), and categorical variables as counts (percentages). Comparisons between groups were performed using the Mann–Whitney U test for continuous variables and the chi-square test for categorical variables. Abbreviations: ACE, angiotensin-converting enzyme; ARB, angiotensin receptor blockers; SGLT2, sodium–glucose cotransporter 2; LVEF, left ventricular ejection fraction; eGFR, estimated glomerular filtration rate; UACR, urinary albumin-to-creatinine ratio; NT-proBNP, N-terminal pro-B-type natriuretic peptide.

Variable	Overall (n = 308)	Non-Frail/Prefrail (n = 155)	Frail (n = 153)	*p* Value
**Baseline parameters**				
Age, years	77 (72–82)	75 (70–80)	79 (74–84)	<0.001
Male sex	220 (71.4%)	121 (78.1%)	99 (64.7%)	0.009
Systolic blood pressure, mmHg	120 (110–130)	—	—	—
Heart rate, bpm	70 (62.5–80)	—	—	—
**Comorbidities**				
Hypertension	275 (89.3%)	140 (90.3%)	135 (88.2%)	0.554
Diabetes mellitus	164 (53.3%)	75 (48.4%)	89 (58.2%)	0.085
Atrial fibrillation	170 (55.2%)	77 (49.7%)	93 (60.8%)	0.050
Ischemic heart disease	141 (45.8%)	78 (50.3%)	63 (41.2%)	0.107
Prior stroke	40 (13.0%)	12 (7.7%)	28 (18.3%)	0.006
Peripheral arterial disease	58 (18.8%)	30 (19.4%)	28 (18.3%)	0.813
Chronic obstructive pulmonary disease	64 (20.8%)	34 (21.9%)	30 (19.6%)	0.615
Liver disease	29 (9.4%)	9 (5.8%)	20 (13.1%)	0.029
Dialysis-dependent chronic kidney disease	37 (12.0%)	10 (6.5%)	27 (17.6%)	0.003
Charlson Comorbidity Index	8 (7–10)	7 (6–8)	9 (8–11)	<0.001
**LVEF category**				0.165
Preserved LVEF	122 (39.6%)	57 (36.8%)	65 (42.5%)	
Mildly reduced LVEF	53 (17.2%)	23 (14.8%)	30 (19.6%)	
Reduced LVEF	133 (43.2%)	75 (48.4%)	58 (37.9%)	
**Medical therapy**				
ACE inhibitors/ARBs	126 (40.9%)	64 (41.3%)	62 (40.5%)	0.891
Sacubitril/valsartan	96 (31.2%)	59 (38.1%)	37 (24.2%)	0.009
Beta-blockers	226 (73.4%)	121 (78.1%)	105 (68.6%)	0.061
SGLT2 inhibitors	240 (77.9%)	124 (80.0%)	116 (75.8%)	0.376
Mineralocorticoid receptor antagonists	123 (39.9%)	74 (47.7%)	49 (32.0%)	0.005
Loop diuretics	192 (62.3%)	87 (56.1%)	105 (68.6%)	0.024
Furosemide	162 (52.8%)	69 (44.8%)	93 (60.8%)	0.005
Torsemide	24 (7.8%)	12 (7.7%)	12 (7.8%)	0.974
Bumetanide	18 (5.8%)	6 (3.9%)	12 (7.8%)	0.137
Thiazides	52 (16.9%)	20 (12.9%)	32 (20.9%)	0.061
Oral anticoagulation	169 (54.9%)	85 (54.8%)	84 (54.9%)	0.984
Vitamin K antagonists	48 (15.6%)	24 (15.5%)	24 (15.7%)	0.961
Direct oral anticoagulants	121 (39.3%)	61 (39.4%)	60 (39.2%)	0.980
**Laboratory parameters**				
Hemoglobin, g/L	130 (118–144)	134 (124–146)	124 (111–141)	<0.001
eGFR, mL/min/1.73 m^2^	34.7 (22.4–51.4)	40.7 (26.3–65.5)	30.0 (19.4–40.6)	<0.001
Albumin, g/L	42.5 (40–45)	43 (42–45)	42 (39–44)	<0.001
Sodium, mmol/L	141 (139–143)	141 (139–143)	140 (138–142)	0.010
Potassium, mmol/L	4.6 (4.2–4.9)	4.6 (4.15–4.9)	4.6 (4.2–4.9)	0.851
UACR, mg/g	43 (11–214)	29 (9–110)	81 (13–264)	0.002
Urinary sodium, mmol/L	59 (40–84)	60.5 (42–87)	59 (39–82.1)	0.275
CA125, U/mL	15 (10–29.5)	12 (8–18)	19 (12–43)	<0.001
NT-proBNP, pg/mL	2420 (1075–6148.5)	1857 (651–3591)	3858 (1621–8750)	<0.001
Ferritin, ng/mL	164 (60–341)	122 (54–256)	200 (79–402)	0.006
Transferrin saturation index, %	22.85 (16.4–31.05)	23.0 (17.1–31.4)	22.7 (15.6–30.6)	0.392

**Table 2:** Baseline Characteristics According to Congestion Status. Patients were categorized according to the presence or absence of congestion. Continuous variables are presented as median (interquartile range), and categorical variables as counts (percentages). Comparisons between groups were performed using the Mann–Whitney U test for continuous variables and the chi-square test for categorical variables. Abbreviations: ACE, angiotensin-converting enzyme; ARB, angiotensin receptor blockers; SGLT2, sodium–glucose cotransporter 2; LVEF, left ventricular ejection fraction; eGFR, estimated glomerular filtration rate; UACR, urinary albumin-to-creatinine ratio; NT-proBNP, N-terminal pro-B-type natriuretic peptide. * Calculated from the sum of individual treatment categories.

Variable	No Congestion	Congestion	*p* Value
**Baseline parameters**			
Age, years	74 (69–80)	78 (73–82)	0.003
Male sex	39 (62.9%)	181 (73.6%)	0.096
Systolic blood pressure, mmHg	120 (120–130)	120 (108–130)	0.065
Heart rate, bpm	70 (60–75)	70 (65–80)	0.003
**Comorbidities**			
Hypertension	56 (90.3%)	219 (89.0%)	0.768
Diabetes mellitus	30 (48.4%)	134 (54.5%)	0.391
Atrial fibrillation	18 (29.0%)	152 (61.8%)	<0.001
Ischemic heart disease	27 (43.5%)	114 (46.3%)	0.693
Prior stroke	5 (8.1%)	35 (14.2%)	0.197
Peripheral arterial disease	8 (12.9%)	50 (20.3%)	0.182
Chronic obstructive pulmonary disease	8 (12.9%)	56 (22.8%)	0.087
Liver disease	5 (8.1%)	24 (9.8%)	0.684
Dialysis-dependent chronic kidney disease	0 (0.0%)	37 (15.0%)	0.001
Charlson Comorbidity Index	6.5 (6–8)	9 (7–10)	<0.001
**LVEF category**			0.391
Preserved LVEF	28 (45.2%)	94 (38.2%)	
Mildly reduced LVEF	12 (19.4%)	41 (16.7%)	
Reduced LVEF	22 (35.5%)	111 (45.1%)	
**Medical therapy**			
ACE inhibitors/ARBs	29 (46.8%)	97 (39.4%)	0.293
Sacubitril/valsartan	24 (38.7%)	72 (29.3%)	0.151
Beta-blockers	48 (77.4%)	178 (72.4%)	0.420
SGLT2 inhibitors	47 (75.8%)	193 (78.5%)	0.653
Mineralocorticoid receptor antagonists	30 (48.4%)	93 (37.8%)	0.128
**Loop diuretics**	17 (27.4%)	145 (59.2%)	<0.001 *
Furosemide	17 (27.9%)	145 (59.2%)	<0.001
Torsemide	2 (3.2%)	22 (8.9%)	0.133
Bumetanide	1 (1.6%)	17 (6.9%)	0.112
Thiazides	8 (12.9%)	44 (17.9%)	0.349
**Oral anticoagulation**	25 (40.3%)	144 (58.5%)	0.011 *
Vitamin K antagonists	9 (14.5%)	39 (15.9%)	0.795
Direct oral anticoagulants	16 (25.8%)	105 (42.7%)	0.015
**Laboratory parameters**			
Hemoglobin, g/L	138 (128–153)	127 (116–142)	<0.001
eGFR, mL/min/1.73 m^2^	48.2 (38.1–74.1)	30.9 (19.9–45.9)	<0.001
Albumin, g/L	44 (42–47)	42 (39–44)	<0.001
Sodium, mmol/L	142 (140–143)	140 (138–142)	0.003
Potassium, mmol/L	4.6 (4.27–4.9)	4.6 (4.15–4.9)	0.587
UACR, mg/g	16.5 (5–51)	54.5 (13–249)	<0.001
Urinary sodium, mmol/L	81 (51–103)	54 (39–80)	<0.001
CA125, U/mL	10 (7–14)	17 (11–37)	<0.001
NT-proBNP, pg/mL	416 (272–713)	3449 (1931–7686)	<0.001
Ferritin, ng/mL	91 (45–201)	183 (72–373)	<0.001
Transferrin saturation index, %	24.0 (19.4–32.7)	22.5 (15.9–30.1)	0.056

**Table 3:** Univariate and Multivariate Cox Regression Analyses for the Composite Outcome of All-Cause Death or Heart Failure Hospitalization at 1 Year. Hazard ratios (HRs) with 95% confidence intervals (CIs) and *p*-values are presented for the association of each clinical and biochemical variable with the primary outcome. Variables with *p* < 0.10 in univariate analyses, together with clinically relevant covariates, were entered into a multivariate Cox model using backward stepwise selection. The final adjusted model retained frailty, congestion, age and sex (male). Abbreviations: HR, hazard ratio; CI, confidence interval; CA125, cancer antigen 125; NT-proBNP, N-terminal pro-B-type natriuretic peptide.

Variable	Univariate HR (95% CI)	*p* Value	Multivariate HR (95% CI)	*p* Value
Frailty	3.00 (1.74–5.17)	<0.001	1.68 (1.00–2.82)	0.047
Congestion	8.98 (2.20–36.70)	0.002	7.44 (1.80–30.78)	0.006
Age (per year)	1.04 (1.00–1.08)	0.042	1.02 (0.98–1.06)	0.407
Male sex	0.81 (0.48–1.36)	0.418	0.83 (0.49–1.40)	0.477
eGFR (per unit)	0.98 (0.97–0.99)	0.007		
Dialysis	2.24 (1.24–4.05)	0.008	—	—
Furosemide	2.38 (1.40–4.07)	0.001	—	—
Albumin (per g/L)	0.88 (0.84–0.94)	<0.001	—	—
Sodium (per mmol/L)	0.96 (0.94–0.98)	<0.001	—	—
CA125 (per U/mL)	1.006 (1.003–1.009)	<0.001	—	—
NT-proBNP (per pg/mL)	1.00003 (1.00001–1.00005)	0.003	—	—
Ferritin (per ng/mL)	1.0007 (1.0003–1.0011)	0.001	—	—

## Data Availability

The data presented in this study are available from the corresponding author upon reasonable request. The data are not publicly available due to privacy and ethical restrictions.

## Supplementary Materials

The following supporting information can be downloaded at: https://www.mdpi.com/article/10.3390/jcm15124715/s1, Table S1: Baseline characteristics according to combined congestion–frailty groups; Table S2: Univariate and multivariate logistic regression analyses for determinants of frailty and congestion; Table S3: Prevalence of congestion according to the original Fried frailty categories; Table S4: Sensitivity analysis using a lower NT-proBNP threshold (≥500 pg/mL) in patients with advanced chronic kidney disease (eGFR 15–45 mL/min/1.73 m^2^); Table S5: Competing-risk analysis for HF hospitalization using a Fine–Gray model.
