# Supplementary material for: Frailty and Congestion in Patients with Heart Failure: Clinical Interaction and Prognostic Implications
Source: J Clin Med. 2026 Jun 17;15(12):4715. doi: 10.3390/jcm15124715 (PMC13301546; doi:10.3390/jcm15124715)
Supplement: Supplementary file 1 [file jcm-15-04715-s001.zip › jcm-4326368-supplementary.pdf]

SUPPLEMENTARY DATA

Supplementary Table S1. Baseline characteristics according to combined congestion–frailty groups. Patients were categorized into four groups based on the presence or absence of clinical congestion and frailty: no congestion/no frailty, congestion without frailty, frailty without congestion, and combined congestion and frailty. Continuous variables are presented as median (interquartile range), and categorical variables as counts (percentages). Comparisons across groups were performed using the Kruskal–Wallis test for continuous variables and the chi-square test for categorical variables. Abbreviations: LVEF, left ventricular ejection fraction; eGFR, estimated glomerular filtration rate; NT-proBNP, N-terminal pro–B-type natriuretic peptide; UACR, urinary albumin-to-creatinine ratio.

Variable	Total (n=308)	No cong / No frag (n=124)	Cong / No frag (n=31)	No cong / Frag (n=82)	Cong / Frag (n=71)	P value
**Baseline parameters**						
Age, years	77 (72–82)	74 (69.5–79)	77 (75–82)	80 (75–84)	78 (74–86)	<0.001
Male sex	220 (71.4%)	98 (79.0%)	23 (74.2%)	57 (69.5%)	42 (59.2%)	0.029
Systolic blood pressure, mmHg	120 (110–130)	120 (110–130)	124 (110–130)	120 (109–135)	120 (108–130)	0.65
Heart rate, bpm	70 (62.5–80)	70 (60–75)	70 (60–80)	70 (65–80)	74 (67–84)	0.010
**Comorbidities**						
Hypertension	275 (89.3%)	111 (89.5%)	29 (93.6%)	70 (85.4%)	65 (91.5%)	0.51
Diabetes mellitus	164 (53.3%)	54 (43.5%)	21 (67.7%)	45 (54.9%)	44 (62.0%)	0.023
Atrial fibrillation	170 (55.2%)	58 (46.8%)	19 (61.3%)	49 (59.8%)	44 (62.0%)	0.11
Prior stroke	40 (13.0%)	11 (8.9%)	1 (3.2%)	17 (20.7%)	11 (15.5%)	0.027
Peripheral arterial disease	58 (18.8%)	22 (17.7%)	8 (25.8%)	11 (13.4%)	17 (23.9%)	0.28
Chronic obstructive pulmonary disease	64 (20.8%)	27 (21.8%)	7 (22.6%)	13 (15.9%)	17 (23.9%)	0.62
Liver disease	29 (9.4%)	7 (5.6%)	2 (6.5%)	6 (7.3%)	14 (19.7%)	0.009
Dialysis	37 (12.0%)	7 (5.6%)	3 (9.7%)	11 (13.4%)	16 (22.5%)	0.006
**LVEF category**						0.414
Preserved LVEF	122 (39.6%)	42 (33.9%)	15 (48.4%)	35 (42.7%)	30 (42.3%)	
Mildly reduced LVEF	53 (17.2%)	19 (15.3%)	4 (12.9%)	15 (18.3%)	15 (21.1%)	
Reduced LVEF	133 (43.2%)	63 (50.8%)	12 (38.7%)	32 (39.0%)	26 (36.6%)	
**Medical therapy**						
ACE inhibitors/ARBs	126 (40.9%)	48 (38.7%)	16 (51.6%)	34 (41.5%)	28 (39.4%)	0.62
Sacubitril/valsartan	96 (31.2%)	54 (43.5%)	5 (16.1%)	23 (28.0%)	14 (19.7%)	0.001
Beta-blockers	226 (73.4%)	98 (79.0%)	23 (74.2%)	54 (65.9%)	51 (71.8%)	0.21
SGLT2 inhibitors	240 (77.9%)	99 (79.8%)	25 (80.6%)	59 (72.0%)	57 (80.3%)	0.51
Mineralocorticoid receptor antagonists	123 (39.9%)	65 (52.4%)	9 (29.0%)	28 (34.1%)	21 (29.6%)	0.003

Variable	Total (n=308)	No cong / No frag (n=124)	Cong / No frag (n=31)	No cong / Frag (n=82)	Cong / Frag (n=71)	P value
Loop diuretics (furosemide)	162 (52.8%)	54 (43.9%)	15 (48.4%)	43 (52.4%)	50 (70.4%)	0.005
Thiazides	52 (16.9%)	13 (10.5%)	7 (22.6%)	10 (12.2%)	22 (31.0%)	0.001
Oral anticoagulation (DOACs)	121 (39.3%)	49 (39.5%)	12 (38.7%)	36 (43.9%)	24 (33.8%)	0.65
Vitamin K antagonists	48 (15.6%)	18 (14.5%)	6 (19.4%)	10 (12.2%)	14 (19.7%)	0.56
**Laboratory parameters**						
Hemoglobin, g/L	130 (118–144)	137 (125–148)	130 (114–137)	126 (118–144)	120 (110–139)	<0.001
eGFR, mL/min/1.73 m²	38.0 (25.8–54.3)	45.3 (34.4–67.1)	31.3 (21.4–47.6)	33.5 (24.9–49.0)	30.8 (19.8–39.9)	<0.001
Albumin, g/L	42.5 (40–45)	43 (42–46)	42 (41–43)	42 (39–44)	42 (38–43)	<0.001
Sodium, mmol/L	141 (139–143)	141 (139–143)	140 (138–143)	140 (139–142)	140 (137–142)	0.029
Potassium, mmol/L	4.6 (4.2–4.9)	4.6 (4.1–5.0)	4.5 (4.2–4.8)	4.6 (4.3–4.9)	4.5 (4.1–5.0)	0.85
Transferrin saturation index, %	22.85 (16.4–31.05)	23.4 (17.1–31.8)	22.6 (15–27.5)	22.9 (16–31)	22.2 (13.9–28.8)	0.52
Ferritin, ng/mL	164 (60–341)	127.5 (54.5–256.5)	91 (51–253)	172 (63–357)	220 (94–450)	0.014
NT-proBNP, pg/mL	2420 (1075–6148.5)	1503 (527.5–3326.5)	3124 (1455–5278)	2763 (1340–7686)	5837 (1977–13516)	<0.001
CA125, U/mL	15 (10–29.5)	11 (7–17.5)	15 (10.8–24)	16 (11–36)	27 (13–62)	<0.001
UACR, mg/g	43 (11–214)	20 (8–95)	46.5 (26–707)	59 (11–264)	98.5 (13.5–264)	<0.001

Supplementary Table S2. Univariate and multivariate logistic regression analyses for determinants of frailty and congestion. Odds ratios (ORs) with 95% confidence intervals (CIs) and p-values are presented for the association of each variable with frailty and congestion. Separate models were constructed for frailty and congestion, with frailty status and congestion status as the respective dependent variables. Variables entered into the multivariable models underwent backward stepwise selection, and only those retained in the final models are shown. Non-frail and non-congested patients served as the reference categories. Abbreviations: OR, odds ratio; CI, confidence interval; LVEF, left ventricular ejection fraction; eGFR, estimated glomerular filtration rate; NT-proBNP, N-terminal pro–B-type natriuretic peptide.

**Variable**	**Frailty Univariate OR (95% CI)**	**p value**	**Frailty Multivariate OR (95% CI)**	**p value**	**Congestion Univariate OR (95% CI)**	**p value**	**Congestion Multivariate OR (95% CI)**	**p value**
Age (per year)	1.08 (1.04–1.12)	<0.001	1.06 (1.03–1.11)	0.001	1.06 (1.02–1.11)	0.006	—	—
Male sex	0.52 (0.31–0.85)	0.010	0.38 (0.21–0.69)	0.002	1.64 (0.91–2.96)	0.098	—	—
Prior stroke	2.67 (1.30–5.47)	0.007	3.37 (1.47–7.72)	0.004	1.89 (0.71–5.05)	0.203	—	—
Liver disease	2.44 (1.07–5.54)	0.033	—	—	1.23 (0.45–3.37)	0.684	—	—
Dialysis	3.11 (1.45–6.67)	0.004	—	—	—	—	—	—
Atrial fibrillation	1.57 (1.00–2.47)	0.051	—	—	3.95 (2.16–7.24)	<0.001	4.15 (1.98–8.70)	<0.001
LVEF (per %)	1.01 (0.99–1.02)	0.380	—	—	0.98 (0.96–1.00)	0.047	0.96 (0.94–0.99)	0.005
eGFR (per unit)	0.97 (0.96–0.98)	<0.001	0.98 (0.96–0.99)	0.001	0.96 (0.95–0.98)	<0.001	0.96 (0.95–0.98)	<0.001
Albumin (per g/L)	0.87 (0.81–0.93)	<0.001	—	—	0.80 (0.72–0.87)	<0.001	0.80 (0.71–0.91)	<0.001
Sodium (per mmol/L)	—	—	—	—	0.88 (0.80–0.97)	0.007	—	—
Potassium (per mmol/L)	0.97 (0.85–1.10)	0.622	—	—	—	—	—	—
Urinary sodium (per mmol/L)	—	—	—	—	0.98 (0.97–0.99)	<0.001	—	—
CA125 (per U/mL)	1.02 (1.01–1.03)	<0.001	1.02 (1.00–1.03)	<0.001	1.09 (1.05–1.14)	<0.001	—	—
Hemoglobin (per g/L)	0.98 (0.97–0.99)	<0.001	—	—	0.98 (0.96–0.99)	0.001	—	—
NT-proBNP (per pg/mL)	1.00 (1.00–1.00)	<0.001	—	—	1.0048 (1.0033–1.0063)	<0.001	—	—
Ferritin	1.00 (1.0000–1.00)	0.001	—	—	1.001 (0.9998–1.0025)	0.057	—	—
Transferrin saturation index	1.00 (0.98–1.01)	0.532	—	—	—	—	—	—
**Congestion**	**4.98 (2.57–9.65)**	**<0.001**	**2.67 (1.24–5.77)**	**0.012**	—	—	—	—
**Frailty**	—	—	—	—	**—**	**—**	**2.66 (1.21–5.83)**	**0.015**

Supplementary Table S3. Prevalence of congestion according to the original Fried frailty categories. A progressive increase in congestion prevalence was observed across the frailty spectrum, from 43.5% in robust patients to 78.9% in prefrail patients and 91.5% in frail patients (p for trend < 0.001), supporting a graded relationship between congestion burden and frailty severity.

Frailty category	No congestion	Congestion	Total
Robust	26 (56.5%)	20 (43.5%)	46
Prefrail	23 (21.1%)	86 (78.9%)	109
Frail	13 (8.5%)	140 (91.5%)	153

Supplementary Table S4. Sensitivity analysis using a lower NT-proBNP threshold (≥500 pg/mL) in patients with advanced chronic kidney disease (eGFR 15–45 mL/min/1.73 m²). Biochemical congestion was alternatively defined using a lower NT-proBNP threshold (≥500 pg/mL) in patients with advanced chronic kidney disease (eGFR 15–45 mL/min/1.73 m²). This approach resulted in the reclassification of 14 patients (4.5% of the cohort), increasing the prevalence of congestion from 79.9% to 84.4%). Odds ratios are adjusted for age and sex. Hazard ratios correspond to Cox proportional hazards models adjusted for age and sex evaluating the composite endpoint of all-cause mortality or heart failure hospitalization.

Outcome	Primary definition	Sensitivity analysis
Prevalence of congestion	79.9%	84.4%
Association with frailty (adjusted OR, 95% CI)	5.23 (2.59–10.59)	5.14 (2.32–11.36)
Association with composite endpoint (adjusted HR, 95% CI)	8.70 (2.12–35.75)	6.36 (1.55–26.18)

Supplementary Table S5. Competing-risk analysis for HF hospitalization using a Fine–Gray model. Fine–Gray subdistribution hazards model evaluating heart failure hospitalization as the event of interest and all-cause mortality as a competing event. The model was adjusted for age and sex.

Variable	Subdistribution HR (95% CI)	P value
Congestion	9.55 (1.30–70.00)	0.026
Age	0.99 (0.93–1.05)	0.798
Male sex	0.65 (0.32–1.31)	0.227
